# Supplementary figures and images for: The SQSTM1-NUP214 fusion protein interacts with Crm1, activates Hoxa and Meis1 genes, and drives leukemogenesis in mice
Source: PLoS One. 2020 Apr 28;15(4):e0232036. doi: 10.1371/journal.pone.0232036 (PMC7188244; doi:10.1371/journal.pone.0232036)

Uncropped blot in Fig 1D

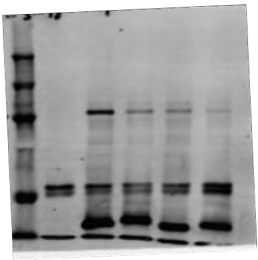

Uncropped blot in Fig 2A

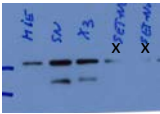

Uncropped blots in Fig 2F

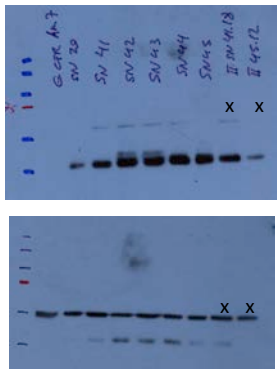

Supplement: S1 Raw images — (PDF) [file pone.0232036.s003.pdf]
